# Enhancing diagnostic preparedness for H5N1: a validation study of H5 single-plex assay and detection across multiple platforms

**DOI:** 10.1128/jcm.00681-25

**Published:** 2025-07-18

**Authors:** Yuan Chao Xue, Jennifer Bertsch, Kaylin Monacy, Carter Haynes, Natalie Williams-Bouyer, Barbara M. Judy, Patrick C. Newman, Thomas G. Ksiazek, Lyudmyla V. Marushchak, Gregory C. Gray, Ping Ren

**Affiliations:** 1 Department of Pathology, University of Texas Medical Branch 198642, Galveston, Texas, USA; 2 Department of Microbiology and Immunology, University of Texas Medical Branch 547647, Galveston, Texas, USA; 3 Department of Internal Medicine, University of Texas Medical Branch 223232, Galveston, Texas, USA; University of California Davis, Davis, California, USA

**Keywords:** H5N1, Clade 2.3.4.4b, LoD, LDT

## Abstract

The increasing transmission of highly pathogenic avian influenza (HPAI) H5N1 from animals to humans underscores the urgent need for enhanced diagnostic capabilities in clinical microbiology laboratories. Although *in silico* analysis suggests that commercial multiplex respiratory panels can detect H5N1, these assays lack subtyping H5N1 capabilities, and their real-world performance remains largely unverified. In this study, we evaluated the limit of detection (LoD) for H5N1 using three commercial molecular diagnostic assays routinely employed at our institution: bioMérieux BioFire Respiratory 2.1 Panel, Cepheid Xpert Xpress CoV-2/Flu/RSV Plus, and Hologic Panther Fusion SARS-CoV-2/Flu A/B/RSV assays. All three reliably detected H5N1 were at low viral concentrations. To enable H5N1 detection, we also developed a real-time RT-PCR H5 single-plex assay on the Hologic Panther Fusion Open Access platform for reflex testing of influenza A-positive specimens. In conclusion, although current commercial assays lack influenza A H5 subtype differentiation, our validation data provide critical performance information. When integrated with a targeted H5 assay, these tools can enhance clinical decision-making and public health surveillance by reducing the risk of missed H5N1 infection cases.

**IMPORTANCE:**

This study addresses a growing public health concern: the spread of bird flu (H5N1) from animals to humans. Most hospital laboratories use commercial tests to detect respiratory viruses like the flu, but these tests cannot tell if someone has the specific and more dangerous H5N1 strain. To help solve this, we tested three commonly used diagnostic tools and found that they can detect H5N1 even at low levels. However, since they cannot identify the specific H5 subtype, we also developed and validated a follow-up test that runs on one of the existing laboratory high-throughput equipment. This test can confirm whether a patient infected with the flu has the H5N1 strain. By combining these tools, hospital laboratories can improve early detection of H5N1, support better patient care, and help public health officials respond more effectively to outbreaks.

## INTRODUCTION

Highly pathogenic avian influenza (HPAI) is a severe and often fatal disease in poultry, primarily caused by influenza A (FluA) viruses of the H5 and H7 subtypes. While wild birds can carry avian influenza viruses, HPAI typically arises within domestic poultry populations. Interactions between wild birds and poultry can facilitate virus transmission and contribute to the emergence and spread of HPAI (1). Sporadic detection of HPAI viruses in a growing number of mammalian species has also been increasingly reported (2). Although human infections have historically been rare, the emergence and global spread of H5N1 clade 2.3.4.4b in wild birds since 2020 has raised significant concerns about cross-species transmission (2). This highly adaptable clade has been sporadically detected in more than 200 mammalian hosts, highlighting its growing zoonotic potential (2, 3). However, sustained transmission has only been documented in a few instances, most notably among dairy cattle in the United States and seals in South America (2, 4–6).

In late March 2024, the first multistate outbreak of H5N1 influenza in dairy cattle was reported in the United States, followed by multiple confirmed cases of animal-to-human transmission (7, 8). In response, the U.S. Centers for Disease Control and Prevention (CDC) recommended expanded H5N1 testing, particularly for hospitalized patients with respiratory illness, and urged clinical laboratories to enhance their capacity to detect non-seasonal influenza viruses (9).

Timely and accurate detection of H5N1 in clinical specimens is critical for effective patient care, outbreak response, and infection prevention. While commercial assay manufacturers have reported that widely used multiplex respiratory assays can detect H5N1 based on *in silico* analysis, these assays do not support subtyping H5, and their diagnostic performance with actual clinical specimens remains largely unverified (10, 11).

To address this gap, clinical laboratories must assess the ability of current molecular diagnostic platforms to detect H5N1 reliably. In this study, we evaluated the analytical sensitivity of three commercial molecular assays routinely used at our institution: bioMérieux BioFire Respiratory 2.1 Panel, Cepheid Xpert Xpress CoV-2/Flu/RSV plus, and Hologic Panther Fusion SARS-CoV-2/Flu A/B/RSV assays. Specifically, we determined the limit of detection (LoD) for H5N1 using inactivated viral isolates. In addition, we took advantage of the Hologic Panther Fusion Open Access platform and developed and validated a real-time RT-PCR H5 single-plex assay. Together, these efforts provide critical performance data to support the use of commercial molecular assays and a laboratory-developed test (LDT) for H5 detection, strengthening laboratory readiness for both clinical care and public health surveillance.

## MATERIALS AND METHODS

### H5N1 virus variants and samples

Four H5N1 virus variants belong to clade 2.3.4.4b of the H5 influenza A virus lineage, A/cattle/Texas/56283/2024 (12) (GenBank accession numbers PP600140–PP600147), A/cattle/Texas/USL_042/2024 (GenBank accession numbers PP914083–PP914090), A/cattle/Texas/MP10/2024 (GenBank accession numbers PP914099–PP914106), and A/Grackle/Texas/USL_047/2024 (GenBank accession numbers PP914091–PP914098) (13), were provided by the World Reference Center for Emerging Viruses and Arboviruses at University of Texas Medical Branch. In addition, five H5N1-positive milk samples were collected from dairy cattle located in New Mexico, USA (12). The A/cattle/Texas/56283/2024 isolate and the five cow milk samples were inactivated by gamma irradiation using Cobalt 60 radiation at a dose of 0.05 MGy. The other three isolates, A/cattle/Texas/USL_042/2024, A/cattle/Texas/MP10/2024, and A/grackle/Texas/USL_047/2024, were inactivated by mixing with TRIzolTM LS reagent at a 1:5 ratio.

### Viral RNA extraction

The A/cattle/USA/Texas/56283/2024 (H5N1) virus stock was diluted in unused BD Universal Viral Transport Medium (UVT) (Franklin Lakes, NJ) prior to RNA extraction. Viral RNA was extracted using the NucleoSpin RNA Virus Extraction Kit (Macherey-Nagel, 740956.10, Düren, Germany), following the manufacturer’s instructions. For A/cattle/Texas/USL_042/2024, A/cattle/Texas/MP10/2024, and A/grackle/Texas/USL_047/2024, viral RNA extraction was performed using Direct-zol RNA Miniprep Plus Kit (Zymo Research, R2072, Irvine, CA), according to the manufacturer’s protocol.

### Viral RNA quantification

H5N1 quantitative qPCR standards, consisting of H5 and N1 gene targets (NZYtech, MD07341, Lisboa, Portugal), were serially diluted in 10-fold, 6 times (2 × 10^6^ copies/µL–2 copies/µL) according to the manufacturer’s instructions. Both extracted H5N1 RNA and diluted quantitative standards were then subjected to RT-qPCR using the influenza A virus H5N1 RT-qPCR kit (NZYtech, MD06641, Lisboa, Portugal), following the recommended protocol. Standard curves were generated based on the RT-qPCR results for the H5 and N1 targets: H5: y = –3.3445x + 39.984 and N1: y = –3.2674x + 40.36. The H5 standard curve was used as the reference for calculating viral titers of the H5N1 variants.

### Diagnostic assays

Three commercial molecular diagnostic assays were evaluated in this study: the BioFire Respiratory Panel 2.1 (bioMérieux, Salt Lake City, UT), the Xpert Xpress CoV-2/Flu/RSV plus assay (Cepheid, Sunnyvale, CA), and the Panther Fusion SARS-CoV-2/FluA/B/RSV assay (Hologic, Marlborough, MA). Diluted virus samples were prepared using remnant Severe acute respiratory syndrome coronavirus 2, influenza A and B, and respiratory syncytial virus (SARS-CoV-2/FluA/B/RSV)-negative Universal Viral Transport (UVT) specimens from routine clinical testing. Samples were then processed on each platform according to the respective manufacturer’s instructions.

### H5 LDT development

Prior to processing and testing on the Panther Fusion system (Marlborough, MA, USA), 500 µL of SARS-CoV-2/FluA/B/RSV-negative UVT sample was transferred into a Specimen Lysis Tube containing 710 µL of specimen transport media. The Internal Control-S and Fusion Capture Reagent-S were automatically added by the Panther Fusion system to each specimen to ensure appropriate specimen processing, amplification, and detection.

The Hologic Open Access Software (Version 2.1) was used to develop an LDT protocol on the Panther Fusion system. The assay utilized the “S” Fusion Capture and Enhancer reagent combination, Hologic RNA internal control primers and probes (Quasar 705 channel), and H5N1-specific primers and probes designed by Sahoo et al. (FAM channel) (14). The optimized primer and probe reconstitution (PPR) composition is shown in Table 1. The thermocycling conditions were as follows: 1 cycle of 50°C for 8 min, 1 cycle of 95°C for 2 min, 1 cycle of 95°C for 2 min, followed by 40 cycles of 95°C for 5 s and 60°C for 30 s. A sample was considered positive if the fluorescence signal crossed 100 Relative Fluorescent Units (RFUs).

**TABLE 1:** Optimized primer and probe reconstitution composition

Components	Sequence 5′→ 3′	Stock concentration	Units	Final concentration
MgCl_2_	–^*b*^	1000	mM	2
KCl	–^*b*^	1000	mM	90
Tris-HCl	–^*b*^	1000	mM	10
Forward Primer #1^*a*^	TAC CAG ATA CTG TCA ATT TAT TCA AC	100	µM	0.4
Reverse Primer #1^*a*^	GTA ACG ACC CAT TGG AGC ACA TCC	100	µM	0.4
Probe #1^*a*^	5(6)-Carboxyfluorescein (FAM)/CTG GCA ATC /ZEN/ ATG RTR GCT GGT CT /3IAbRQSp/	100	µM	0.2
Forward Primer #2^*a*^	TGG GTA CCA TCA TAG CAA TGA GCA	100	µM	0.4
Reverse Primer #2^*a*^	AAC TCC CTT CCA ACT GCC TCA AA	100	µM	0.4
Probe #2^*a*^	5(6)-Carboxyfluorescein (FAM)/TGG GTA CGC /ZEN/ TGC GGA CAA AGA ATC CA /3IAbRQSp/	100	µM	0.2
Hologic RNA IC Primers	–^*c*^	37.5	µM	0.6
Hologic RNA IC Probes	–^*c*^	25	µM	0.4

^*a*^ Primers and probes target only the HA gene in influenza A.

^*b*^ “–” in the upper three rows (MgCl₂, KCl, Tris-HCl) indicate not applicable*.*

^*c*^ “–” in the lower two rows (Hologic RNA IC primers and probes) indicate data not available due to proprietary restrictions (Hologic Inc.).

### H5 LDT assay validation:

The LoD of the H5 LDT assay was assessed using eight dilutions of A/cattle/USA/Texas/56283/2024 isolate, each tested in at least 10 replicates, prepared in SARS-CoV-2/FluA/B/RSV-negative UVT. Analytical specificity and potential cross-reactivity were evaluated using 51 FluA-negative clinical specimens that were positive for other common viral and bacterial respiratory pathogens and 134 FluA-positive clinical specimens collected since 2022 (Table S2). To assess accuracy, 47 contrived specimens were prepared by spiking inactivated H5N1 virus into pooled negative UVT at two concentrations: 5.8 × 10^4^ RNA copies/mL (25 samples) and 2.9 × 10^4^ RNA copies/mL (22 samples). Each sample was tested in quintuplicate over five consecutive days. Repeatability (within-run precision) was measured by testing three replicates of specimens at four different viral concentrations. Reproducibility (inter-run, inter-day precision) was assessed by testing negative, near-LoD (1.45 × 10^4^ RNA copies/mL), low (2.9 × 10^4^ RNA copies/mL), and high (5.8 × 10^5^ RNA copies/mL) viral concentrations in triplicate by two independent operators over three consecutive days.

### Statistical analysis

Standard curve generation and viral quantification for H5N1 were performed using Microsoft Excel (Version 2501). Probit analysis, sensitivity, specificity, and 95% confidence interval (CI) were conducted using MedCalc (Version 23.1.7) (https://www.medcalc.org/calc/). Statistical analysis and data visualization for the H5 LDT reproducibility and accuracy studies were performed by Prism 8 (Version 8.0.2).

## RESULTS

**Fig 1:**
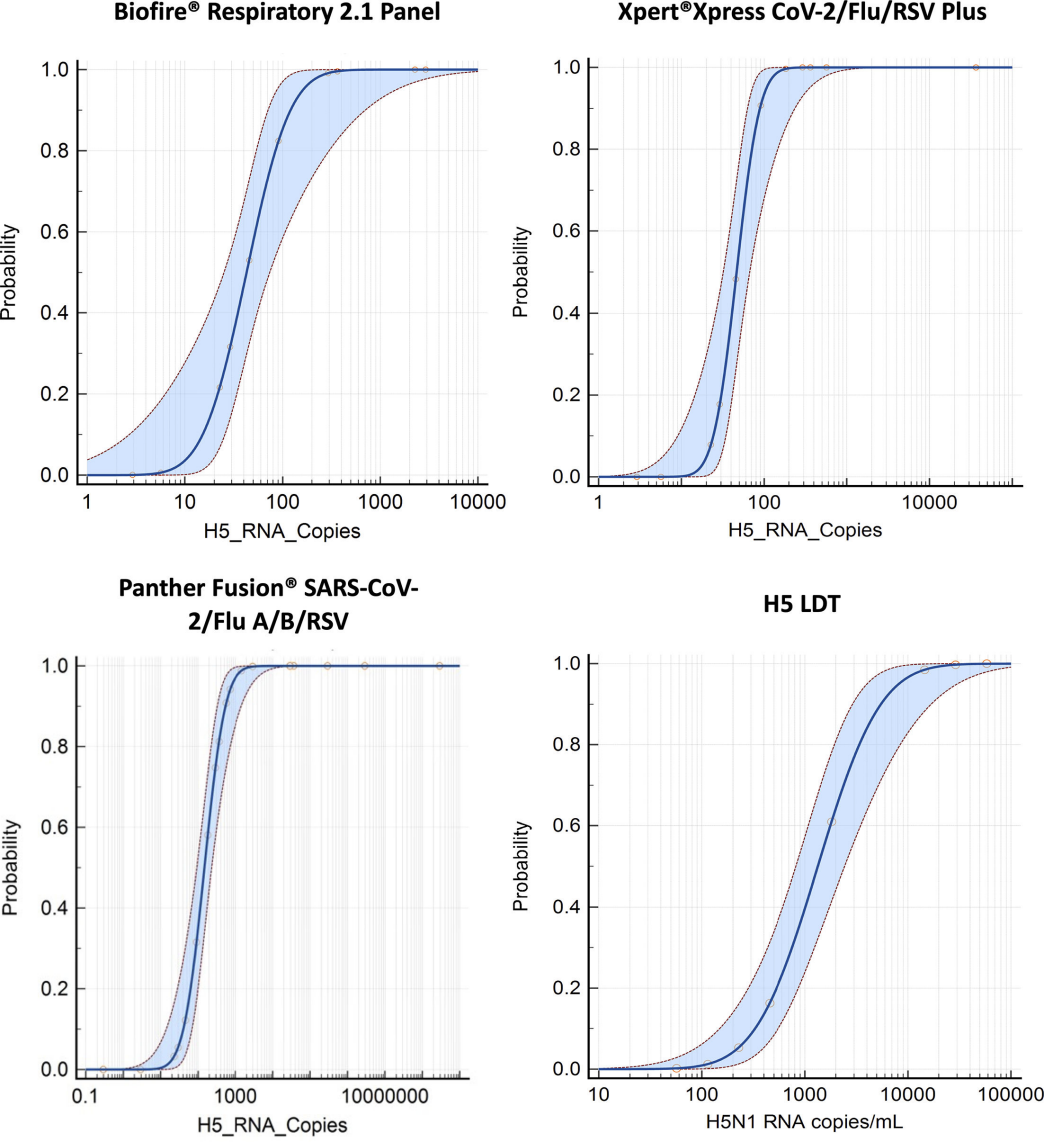
Probit analysis of LoD for Influenza A H5N1 on three commercial molecular diagnostic assays and the H5 LDT assay. Probit regression was used to determine the LoD of H5N1 using A/cattle/USA/Texas/56283/2024 for each assay. The x-axis represents viral RNA copies/mL, and the y-axis indicates the probability of detection. The LoD is defined as the viral RNA concentration corresponding to a 95% detection probability. The red circles are data points (dilutions tested) that represent the observed detection rates for each dilution, and the fitted probit curves represent the statistical modelling of detection probabilities. Shaded areas show the 95% CI around the probit curves.

The LoD at a 95% CI was determined for the three commercial molecular diagnostic assays and the H5 LDT using the A/cattle/Texas/56283/2024 variant. The estimated LoD values for each assay are summarized in Table 2. Fig. 1 presents the probit analysis results, with the LoD defined as the viral RNA concentration corresponding to a 95% probability of detection (*P* < 0.0001).

**TABLE 2:** LoD of three commercial diagnostic assays for influenza A and an H5 LDT

Manufacture	Assay	LoD (RNA copies/mL)	95% CI (RNA copies/mL)
bioMérieux	BioFire Respiratory Panel 2.1	161.77	87.49–1444.49
Cepheid	GeneXpert Xpress CoV-2/Flu/RSV plus	107.03	72.38–354.31
Hologic	Panther Fusion SARS-CoV-2/FluA/B/RSV	783.66	436.99–2579.84
Hologic	H5 LDT	8174.48	4017.17–30140.97

To optimize the H5 LDT, various PPR buffer compositions were tested. Final concentrations of KCl (0, 30, 60, and 90 mM), MgCl_2_ (2, 3, 4, and 5 mM), and Tris-HCl (4, 6, 8, and 10 mM) were evaluated to identify conditions that produced consistent Ct values with minimal background amplification when using a positivity threshold at 100 RFU (Table S1). The finalized optimized concentrations used in the assay are detailed in Table 1.

Using the optimized PPR conditions, analytical specificity was evaluated using 185 nasopharyngeal swab (NPS) samples in UVT, comprising samples positive for 15 common respiratory viruses and 3 bacterial pathogens. No cross-reactivity was observed with any non-H5N1 targets (Table S3). The LDT assay accurately detected all 47 spiked samples containing inactivated H5N1 virus. Day-to-day testing over five consecutive days showed consistent Ct values with no significant intra- or inter-day variation (Fig. S1A). Reproducibility testing conducted by two independent operators across three days confirmed consistent performance with no significant inter-day or inter-operator variability (Fig. S1B). Additionally, minimal test-to-test variability was observed in multiple PCR replicates of the same H5N1-spiked sample (Fig. S1C). Overall, the LDT demonstrated 100% sensitivity (95% CI: 92.45%–100%) and 100% specificity (95% CI: 98.03%–100%) (Table S3).

To further validate the detection capability of three commercial platforms and the H5 LDT, three additional H5N1 variants, A/cattle/Texas/USL_042/2024, A/cattle/Texas/MP10/2024, and A/grackle/Texas/USL_047/2024, were tested using either diluted virus or viral RNA spiked into UVT. All three commercial assays and the LDT successfully detected each variant (Table 3). The LDT also reliably detected H5N1 in five raw cow milk specimens previously confirmed as positive (Table 3).

**TABLE 3:** Detection of other H5N1 variants and samples

	bioMérieux BioFire	Cepheid GeneXpert	Hologic Panther Fusion	H5 LDT^1*a*^
**Specimen types**	**Results**	**Ct values**	**Ct values**	**Ct values**
A/cattle/Texas/MP10/2024	Equivocal^*b*^	31.8	37.3	30.4
A/cattle/Texas/USL_042/2024	Detected^*b*^	31.2	35.3	28
A/grackle/Texas/USL_047/2024	Equivocal^*b*^	31.1	36.3	32.63
Milk 1	–^*c*^	–	–	19.1
Milk 2	–	–	–	21.8
Milk 3	–	–	–	23.8
Milk 4	–	–	–	26.9
Milk 5	–	–	–	21.7

^*a*^ LDT: Laboratory Developed Test.

^*b*^ Equivocal: FluA-pan-1 or FluA-pan-2 was positive but FluA-H1-2, FluA-H1-2009, and FluA-H3 were negative; Detected: Both FluA-pan-1 and FluA-pan-2 were positive but FluA-H1-2, FluA-H1 2009, and FluA-H3 were negative.

^*c*^ "–”, indicates that the experiment was not performed.

## DISCUSSION

Although H5N1 was first identified in a Scottish poultry farm in 1959, it did not emerge as a significant public health concern until the 1997 outbreak in Hong Kong, which resulted in 18 confirmed human infections and six deaths (15). Since then, ongoing circulation and genetic reassortment with other influenza A viruses have led to the emergence of multiple distinct H5N1 clades. The current strain of concern belongs to clade 2.3.4.4b, which initially spread among birds in Europe and subsequently adapted to more efficiently infect wild bird populations (15). Genetic divergence between the 1997 Hong Kong strain and the currently circulating clade 2.3.4.4b variant is substantial and continues to expand as the virus evolves. This increasing genetic variability poses challenges for molecular diagnostics, as many assays developed for earlier strains may demonstrate reduced sensitivity or variable performance when detecting contemporary clade 2.3.4.4b variants (14).

To account for genetic variability, many molecular assays are designed to target multiple gene regions (16). For example, the Cepheid Xpert Xpress CoV-2/Flu/RSV Plus assay detects three targets: the matrix (M) gene and two viral polymerase genes, PB2 and PA (17). In contrast, the Hologic Panther Fusion SARS-CoV-2/Flu A/B/RSV assay targets only the M gene (18). The bioMérieux BioFire Respiratory 2.1 Panel does not disclose specific gene targets but includes two pan-influenza markers and three hemagglutinin-based subtyping targets to differentiate H1, H1-2009, and H3 (19).

The H5 LDT assay presented in this study uses primers and probes developed by Sahoo et al., which specifically target the HA gene of clade 2.3.4.4b H5N1 variants (14). Unlike proprietary commercial assays, these primer and probe sequences were modified from the original World Health Organization (WHO) design to improve inclusivity for 2.3.4.4b strains while simplifying assay complexity (14, 20). Ultimately, both the number of gene targets and the specific design of primers and probes significantly influence assay performance. Sequence alignment analysis revealed 14–17 base mismatches between each of the four H5N1 variants tested and each of the four primers used in the H5 LDT assay (Fig. S2). The distribution of these mismatches across the HA gene varied by variants, with the exception of primer #1 forward and primer #2 reverse, which showed conserved mismatch patterns (Fig. S2). This heterogeneity highlights the robustness of the primer and probe design in accommodating sequence diversity among circulating H5N1 clade 2.3.4.4b variants.

The decision to implement the H5 LDT on the Hologic Panther Fusion Open Access platform was driven by both operational and practical considerations, despite its relatively higher LoD compared to other platforms. The Panther Fusion system is widely adopted in clinical microbiology laboratories and supports high-throughput, fully automated molecular testing with flexible assay scheduling and minimal hands-on time. Its Open Access functionality allows laboratories to rapidly develop and deploy LDTs using custom primer/probe sets without the need for additional instrumentation. Additionally, the overall cost of implementation is relatively lower than that of other platforms. These advantages made Pather Fusion an ideal choice for integrating a reflex subtyping assay for H5N1 into existing respiratory diagnostic workflows.

Nonetheless, technical aspects such as gene target selection, primer/probe design, and assay architecture also influence analytical sensitivity, contributing to differences observed among platforms and across influenza A subtypes. For the Panther Fusion SARS-CoV-2/Flu A/B/RSV assay, the reported LoDs using NPS matrix are 0.06 TCID_50_/mL for H1N1 (2018) and 0.11 TCID_50_/mL for H3N2 (2017) (18). For the Xpert Xpress CoV-2/Flu/RSV Plus assay, the reported LoDs using NPS matrix are 0.007 TCID_50_/mL for H1N1 (Idaho/2018), 0.0022 TCID_50_/mL for H1N1 (California/2009), 0.44 TCID_50_/mL for H3N2 (Hong Kong/2019), and 0.05 TCID_50_/mL for H3N2 (Victoria/2011) (17). For the Biofire Respiratory 2.1 Panel, the reported LoDs using NPS matrix are 1000 TCID_50_/mL for H1N1 (1999), 0.5 TCID_50_/mL for H1N1pdm09 (2009), and 0.1 TCID_50_/mL for H3N2 (1973) (19). In our study, the detection thresholds for H5N1 were 107.03 RNA copies/mL (equivalent to 0.11 TCID_50_/mL) for the Xpert Xpress CoV-2/Flu/RSV Plus assay, 161.77 RNA copies/mL (equivalent to 0.16 TCID_50_/mL) for the Biofire Respiratory 2.1 Panel, and 783.66 RNA copies/mL (equivalent to 0.78 TCID_50_/mL) for the Panther Fusion SARS-CoV-2/Flu A/B/RSV assay. RNA copy numbers were converted to TCID_50_/mL using an established approximation of 1000 influenza RNA copies/mL per 1 TCID_50_/mL (21, 22). These findings highlight assay-specific differences in sensitivity that extend beyond H5N1 detection, potentially impacting the broader clinical utility of each platform.

Building on the observed variability, the difference in LoDs between those achieved by Sahoo et al. (<0.5 RNA copies/µL or <500 RNA copies/mL) and our H5 LDT (8174.48 RNA copies/mL) is likely attributable to several methodological differences. These include the real-time RT-PCR platforms utilized (Invitrogen versus Hologic), the nature of the positive control materials (synthetic single-stranded DNA versus inactivated whole virus), and the specimen preparation methods (naked nucleic acids versus inactivated virus in UVT matrix). Notably, the approach used in this study more closely represents real-world clinical conditions, in which viral nucleic acids must be extracted from NPS prior to RT-PCR amplification, an additional step that can reduce the overall sensitivity of the assay.

In addition to differences in assay sensitivity across studies, there are also notable variations in the LoDs for our H5 LDT compared to the three commercial diagnostic platforms. These differences are likely due to factors such as the number and selection of gene targets, as well as the specific primers and probes used in the assays. This discrepancy is particularly relevant when the H5 LDT is used to screen all FluA-positive specimens that have already tested positive on a commercial multiplex assay, as the higher LoD of the H5 LDT increases the risk of false-negative subtyping results. Therefore, if this workflow is adopted, it is essential to consider this limitation when interpreting borderline or unexpected findings.

Another limitation of this H5 LDT validation is the absence of confirmed H5N1-positive clinical samples. All positive samples used in the study were contrived, designed to mimic clinical specimens by spiking inactivated virus or extracted RNA into the pooled negative matrix. While the inclusion of H5N1-positive raw cow milk specimens provided additional insight into the assay’s performance, this matrix differs substantially from human respiratory samples. Despite these limitations, the contrived samples represented the best available materials for assay development and validation at the time of study.

### Conclusion

Although three commercial assays do not provide influenza A H5 subtyping, their reliable detection of H5N1 in the pan-FluA callout at low viral concentrations, in combination with the validated H5 LDT, supports their utility in frontline diagnostic workflows. As H5N1 continues to pose a potential public health threat, ensuring high accuracy and sensitivity across widely accessible molecular platforms remains critical for effective surveillance and containment. This validation study provides key data to support laboratory decision-making in assay selection and implementation, ultimately strengthening diagnostic readiness for emerging influenza threats and enabling timely patient care and outbreak response.

## SUPPLEMENTAL MATERIAL

Supplemental figures and tables jcm.00681-25-s0001.docx Fig. S1 and S2, and Tables S1 to S3.

ASM does not own the copyrights to Supplemental Material that may be linked to, or accessed through, an article. The authors have granted ASM a non-exclusive, world-wide license to publish the Supplemental Material files. Please contact the corresponding author directly for reuse.
