# Supplementary material for: Enhancing diagnostic preparedness for H5N1: a validation study of H5 single-plex assay and detection across multiple platforms
Source: J Clin Microbiol. 2025 Jul 18;63(8):e00681-25. doi: 10.1128/jcm.00681-25 (PMC12345207; doi:10.1128/jcm.00681-25)
Supplement: Supplemental figures and tables — Fig. S1 and S2, and Tables S1 to S3. [file jcm.00681-25-s0001.docx]

**
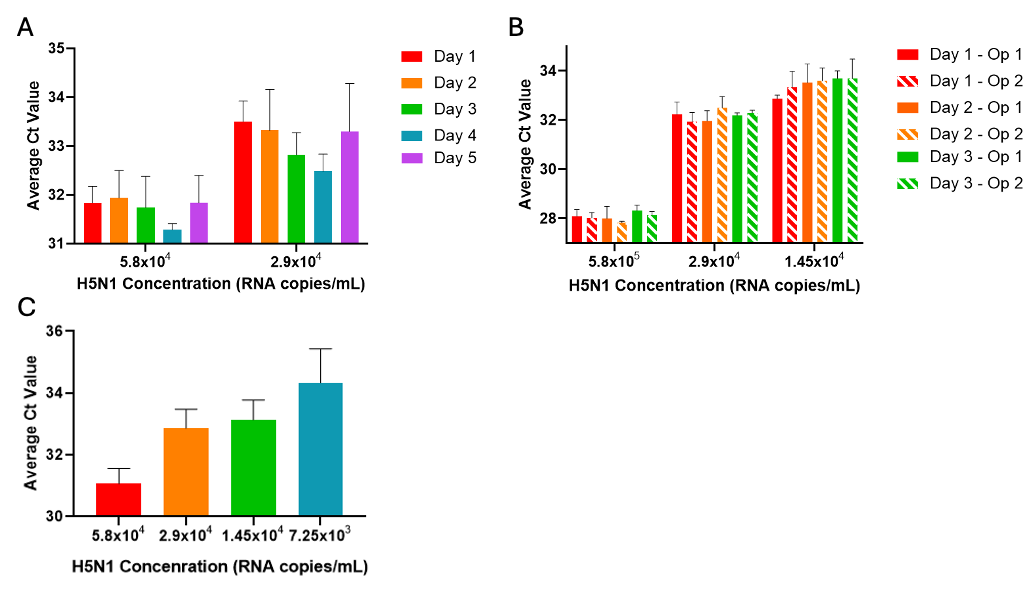
**

**Supplementary Figure 1:** Accuracy, reproducibility, and repeatability of H5 LDT assay. The x-axis represents the spiked H5N1 concentration in RNA copies/mL, and the y-axis represents the average Ct values. Each bar represents the average Ct values per day (A: accuracy), per day per operator (B: reproducibility) or per specific H5N1 concentration (C: repeatability), along with the corresponding standard deviation values of each dataset. One-way ANOVA statistical analysis was performed for the accuracy data, while two-way ANOVA statistical analysis was performed for the repeatability data, and neither showed statistically significant differences (p>0.05).

**
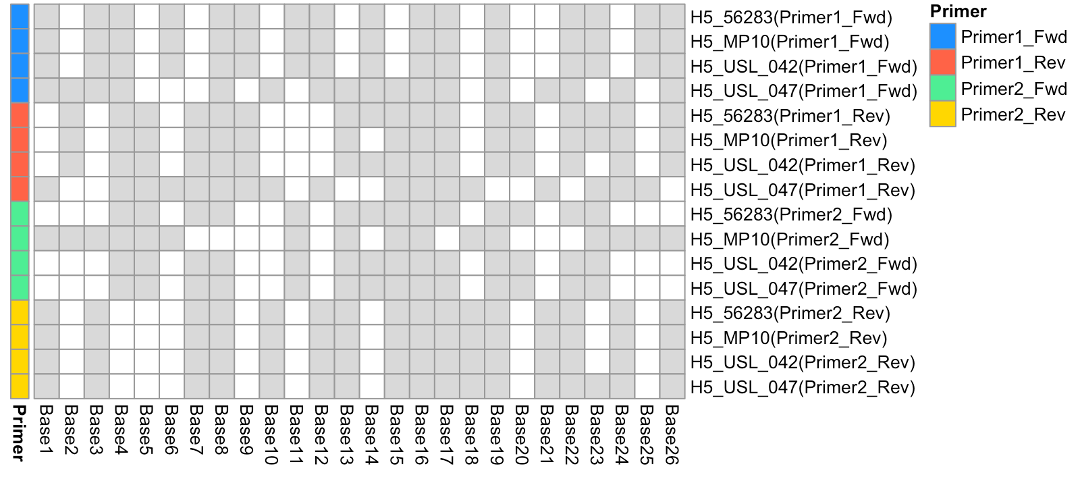
**

**Supplementary Figure 2:** Per-base mismatches for all H5 LDT primers. The x-axis represents each base/nucleotide on the primers, while the y-axis displays the match between the specific H5N1 variant’s *HA* gene and the primer. A gray box indicates a mismatch between the base of the primer and the corresponding nucleotide of the H5N1 variant’s *HA* gene, while a white box indicates a match.

**Supplementary Table 1:** Ct Values of H5 LDT PPR Optimizations

|  | **4 mM MgCl_2_ and 10 mM Tris-HCl** | | | | |  |  |  | |  |
| --- | --- | --- | --- | --- | --- | --- | --- | --- | --- | --- |
| **KCl (mM)** | **0** | | **30** | **60** | **90** |  |  |  | |  |
| **Controls** | **Pos** | **Neg** | **Pos** | **Pos** | **Pos** |  |  |  | |  |
| **PCR replicates** | 36 | 14 | 27.1 | 30.2 | 31.2 |  |  |  | |  |
|  | 36.1 | 13.7 | 28.3 | 30 | 31.5 |  |  |  | |  |
|  | 36 | 14.3 | 26.8 | 30.3 | 31.5 |  |  |  | |  |
| **Average** | 36.0 | 14.0 | 27.4 | 30.2 | 31.4 |  |  |  | |  |
|  |  |  |  |  |  |  |  |  | |  |
|  | **60 mM KCl and 10 mM Tris-HCl** | | | | **90 mM KCl and 10 mM Tris-HCl** | | | | | |
| **MgCl_2_ (mM)** | **2** | **3** | **4** | **5** | **2** | **3** | **4** | | **5** | |
| **Controls** | **Pos** | **Pos** | **Pos** | **Pos** | **Pos** | **Pos** | **Pos** | | **Pos** | |
| **PCR replicates** | 31 | 31.3 | 29.9 | 30.8 | 32.1 | 31.5 | 31.6 | | 31.5 | |
|  | 30.5 | 30.6 | 30.6 | 31.2 | 32.3 | 31.9 | 31.9 | | 31.9 | |
|  | 31.2 | 30.7 | 30.5 | 30.8 | 31.5 | 31.6 | 30.8 | | 32 | |
| **Average** | 30.9 | 30.9 | 30.3 | 30.9 | 32.0 | 31.7 | 31.4 | | 31.8 | |
|  |  |  |  |  |  |  |  |  | |  |
|  | **2 mM MgCl_2_ and 90 mM KCl** | | | | **4 mM MgCl_2_ and 90 mM KCl** | | | | | |
| **Tris-HCl (mM)** | **4** | **6** | **8** | **10** | **4** | **6** | **8** | | **10** | |
| **Controls** | **Pos** | **Pos** | **Pos** | **Pos** | **Pos** | **Pos** | **Pos** | | **Pos** | |
| **PCR replicates** | 31.2 | 30.5 | 31.5 | 31.6 | 32.2 | 32.1 | 31.1 | | 31.6 | |
|  | 31.2 | 31.2 | 30.8 | 31.3 | 31.8 | 31 | 31.4 | | 30.9 | |
|  | 31.1 | 31.9 | 31.2 | 31.5 | 32.4 | 31.9 | 31.9 | | 31.2 | |
| **Average** | 31.2 | 31.2 | 31.2 | 31.5 | 32.1 | 31.7 | 31.5 | | 31.2 | |

**Supplementary Table 2:** List of H5-Negative Clinical Specimens

| **Pathogens Detected in Clinical Specimens** | **# of Positives Used** | **Was H5 detected (Yes/No)** |
| --- | --- | --- |
| Adenovirus | 6 | No |
| Coronavirus 229E | 3 | No |
| Coronavirus HKU1 | 4 | No |
| Coronavirus NL63 | 3 | No |
| Coronavirus OC43 | 4 | No |
| SARS-CoV-2 | 3 | No |
| Human metapneumovirus | 5 | No |
| Human rhinovirus/enterovirus | 15 | No |
| Influenza A virus | 93 | No |
| Influenza A virus A/H3 | 15 | No |
| Influenza A virus A/H1-2009 | 32 | No |
| Influenza B virus | 3 | No |
| Parainfluenza virus 1 | 3 | No |
| Parainfluenza virus 2 | 3 | No |
| Parainfluenza virus 3 | 4 | No |
| Parainfluenza virus 4 | 4 | No |
| Respiratory Syncytial Virus (RSV) | 5 | No |
| *Bordetella parapertussis* | 2 | No |
| *Bordetella pertussis* | 3 | No |
| *Mycoplasma penumoniae* | 2 | No |

**Supplementary Table 3**: Analytical Sensitivity and Specificity of H5 LDT

|  |  | **UVT^1^ Specimens** | |  |
| --- | --- | --- | --- | --- |
|  |  | **Positive** | **Negative** | **Total** |
| **H5 LDT^1^** | **Positive** | 47 | 0 | 47 |
|  | **Negative** | 0 | 185 | 185 |
|  | **Total** | 47 | 185 | 232 |
|  | **Sensitivity (95% CI^1^)** | 100% (93.15% - 100%) |  |  |
|  | **Specificity (95% CI)** |  | 100% (98.03% - 100%) |  |

^1^ UVT: BD^TM^ Universal Viral Transport Medium; LDT: Laboratory Developed Test; CI: Confidence Interval
